# Surgical Removal of Neglected Soft Tissue Foreign Bodies by Needle-Guided Technique 

**Published:** 2013

**Authors:** Ali Ebrahimi, Mohammad Radmanesh, Sohrab Rabiei, Hossein kavoussi

**Affiliations:** 1 *Department of Dermatology, Kermanshah University of Medical Sciences, Kermanshah, Iran.*; 2 *Department of Dermatology, Jondishapour University of Medical Sciences, Ahwaz, Iran.*; 3 *Department of Otolaryngology, Kermanshah University of Medical Sciences, Kermanshah, Iran.*

**Keywords:** Foreign body, Soft tissue, Surgery

## Abstract

**Introduction:**

The phenomenon of neglected foreign bodies is a significant cause of morbidity in soft tissue injuries and may present to dermatologists as delayed wound healing, localized cellulitis and inflammation, abscess formation, or foreign body sensation. Localization and removal of neglected soft tissue foreign bodies (STFBs) is complex due to possible inflammation, indurations, granulated tissue, and fibrotic scar. This paper describes a simple method for the quick localization and (surgical) removal of neglected STFBs using two 23-gauge needles without ultrasonographic or fluoroscopic guidance.

**Materials and Methods:**

A technique based on the use of two 23-gauge needles was used in 41 neglected STFBs in order to achieve proper localization and fixation of foreign bodies during surgery.

**Results:**

Surgical removal was successful in 38 of 41 neglected STFBs (ranging from 2–13mm in diameter).

**Conclusion:**

The cross-needle-guided technique is an office-based procedure that allows the successful surgical removal of STFBs using minimal soft tissue exploration and dissection via proper localization, fixation, and propulsion of the foreign body toward the surface of the skin.

## Introduction

Up to 38% of soft tissue foreign bodies (STFBs) are neglected in initial clinical examinations in emergency departments, and 25% of all STFBs are presented weeks, months, and even years after a penetrating injury (1,2,3). Numerous strategies and imaging modalities have been reported in multiple studies to define an accurate method for the detection of STFBs, but the phenomenon of neglected foreign bodies remains a significant cause of morbidity in soft tissue injuries. The likelihood of STFB oversight depends upon the presence of tenderness, swelling, and hematoma following injury as well as on the nature, size, location, and number of foreign bodies (4,5). The use of standard, sensitive, and specific localizing equipment and an experienced operator are important in STFB detection (6,7).

Patients with skin and soft tissue penetrating injuries and suspected foreign bodies commonly present to the emergency department for evaluation and treatment. However, neglected foreign bodies may present to dermatologists simply as delayed wound healing, localized cellulitis and inflammation, abscess formation, or foreign body sensation (8-11). Many dermatologists are also involved in scar revision or laser procedures for scars and traumatized tattoos in this field. The most important step in the removal of a neglected STFB is accurate localization, followed by perfect incision and dissection of an old scar to identify the foreign body surrounded by fibrotic scar tissues. 

Ultrasonography is the first-choice technique for clinicians investigating the presence of STFBs. Fluoroscopic- or computed-tomographic-guided foreign body removal are used only for opaque STFBs as these techniques expose the patient and operators to radiation and require expensive equipment (12-14). An office-based approach involves making an elliptical incision around the entry wound and removing the foreign body from the surrounding skin and soft tissues (4). However, this is difficult to perform in oblique-penetrated or deep embedded STFBs and can lead to scar formation in cosmetically important areas. Incision over the foreign body and blind dissection is another office-based technique, but the exact position of foreign bodies in soft tissues is usually not evident. 

Here we describe a needle-guided technique as an office-based, cost-effective and time-saving technique that reliably facilitates localization and removal of the neglected STFBs in a very simple and efficient manner. In this approach, needles are used to quickly localize and fix the foreign body and propel it towards the surface of the skin, where foreign body removal is performed using minimal soft tissue exploration and dissection.

## Materials and Methods

Fifteen patients aged between 10 and 54 years, including six males and nine females, presented at our clinic between March 2006 and August 2010. Patient complaints included foreign-body sensation, local inflammation at the site of an old scar, and requests for laser procedures for traumatized tattoos and scars. Three males and one female, aged 22–54 years, were injured during and after the Iran–Iraq war (1980–1988) by a bomb or mine explosion. One 32-year old woman received a pen injury at school 20 years earlier, while all other subjects were injured in car accidents. These injuries had taken place 1–24 (mean, 10.44) years ago.

Among 41 foreign bodies detected by physical examination, 10 were located in periorbital area and eight in the forehead, with others located in the cheek, upper lip, forearm, hand, or finger. Preoperative neurologic and functional examinations of the affected areas were performed to detect any damage to nerves, tendons, or muscles that may have occurred during injury. Previous medical and medication histories were also noted.

Written informed consent was obtained from all patients and the father of a 10-year old boy. The study protocol conformed to the guidelines of the 1975 Declaration of Helsinki. Our Institutional Review Board and Ethics Committee approved this study.

**Fig 1:**
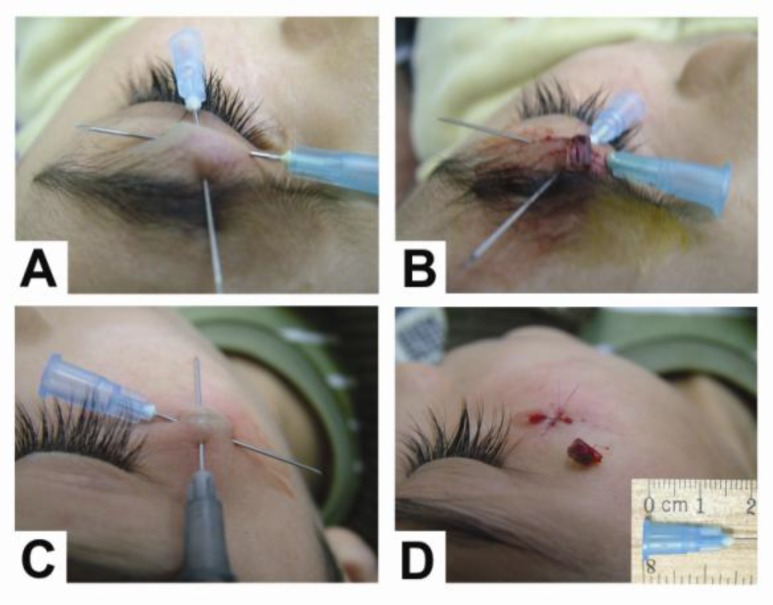
A and B, Localization and removal of an upper eyelid foreign body in a 10-year old boy 2 years after a car accident; C and D, Removal of a cheek foreign body in the same patient

*Technique: *Topical anesthesia, Eutectic Mixture of Local Anesthetics (EMLA) cream, was applied 30–60 min before surgery. After palpating carefully for foreign bodies or a point of maximum tenderness, the first 23-gauge needle tip was inserted into the skin through a point 5–10 mm away from the foreign body site and was advanced below the foreign body. Greater upward and forward pressure to the end of the needle led to the emergence of the needle tip from the other side of the foreign body (Fig 1). Next, the surgical field was anesthetized using 2% lidocaine with 1/100,000 epinephrine or digital nerve/field blocks with 1% plain lidocaine. Then, a second 23-gauge needle was inserted across the first needle and below the foreign body in the same manner to help localize the foreign body and propel it towards the surface of the skin (Fig 1B and C).

**Fig 2:**
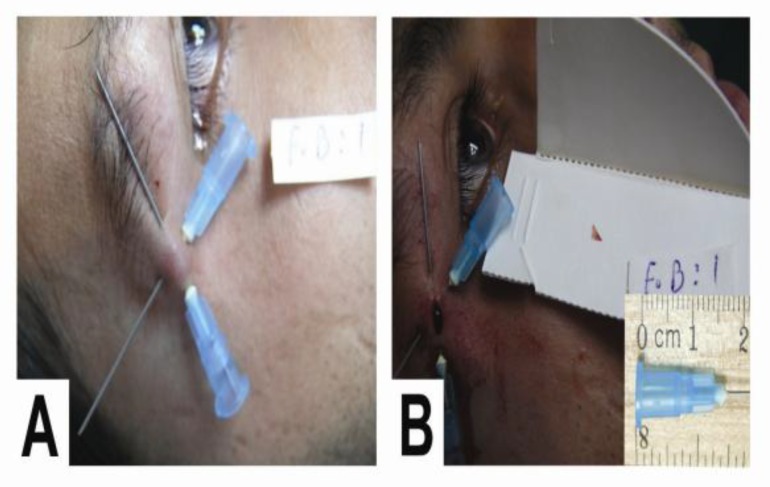
Localization and removal of a small piece of glass in a 24-year old man with complaints of tenderness in the lateral end of his eyebrow 1 year after a car accident

As shown in Fig 1, needle tip was located some distance away from the orbital area to prevent any potential trauma to the globe. A bloodless field was achieved using lidocaine with epinephrine or by placing a tourniquet proximal to the incision. Once localization was achieved, an incision was made directly over the fixed foreign body using a number-15 scalpel blade. Finally, by minimal blunt dissection, the foreign body was released from the surrounding tissue and removed from the body (Fig 2). The wounds were then examined and irrigated using isotonic saline solution. Wounds were repaired using separate or vertical mattress nylon sutures. All patients received oral antibiotics for 5–7 days after the procedure, and sutures were removed 5–7 days after surgery. Post-operative neurologic and functional assessment of the affected area was performed to detect complications of STFB removal.

## Results

Surgical removal was successful in (38 of 41) cases of neglected STFBs. Thirty-four of the foreign bodies successfully removed were approximately 2–5 mm in diameter (Fig 2), including 27 small pieces of glass, five stones, and two small metal objects. The biggest object was a 13-mm (stone, which) had been neglected in the forearm of a (soldier) after a mine explosion.

All other three STFBs removed were 5–10 mm in diameter. 

**Fig 3:**
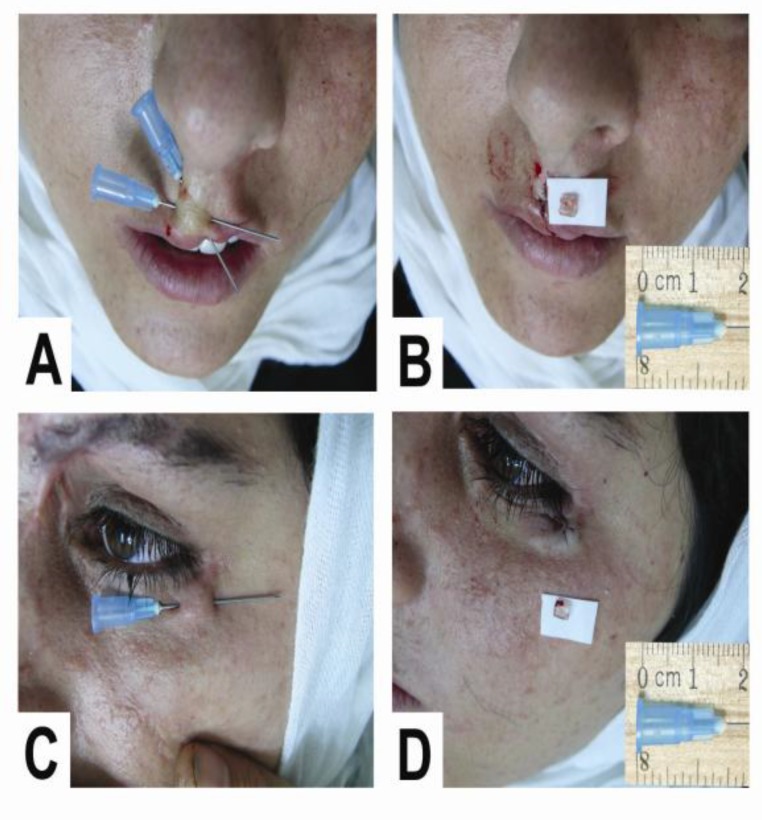
Removal of an STFB in a 27-year old woman with upper lip and lower lid foreign body sensation 14 months after a car accident, with multiple traumas and several facial scars. Radiographs obtained were negative

Plain radiographs were obtained from all patients before the operation, 12 of which were negative even though neglected SFTBs were actually present in the hand, forehead, perioral, and periorbital area. In nine cases, the STFBs which gave a negative radiograph were small pieces of glass with a diameter of 2–3 mm (Fig 3B and D).

Surgical removal was unsuccessful in the case of three foreign bodies with a negative radiograph, located in the periorbital area and the dorsum of the hand. 

**Fig 4:**
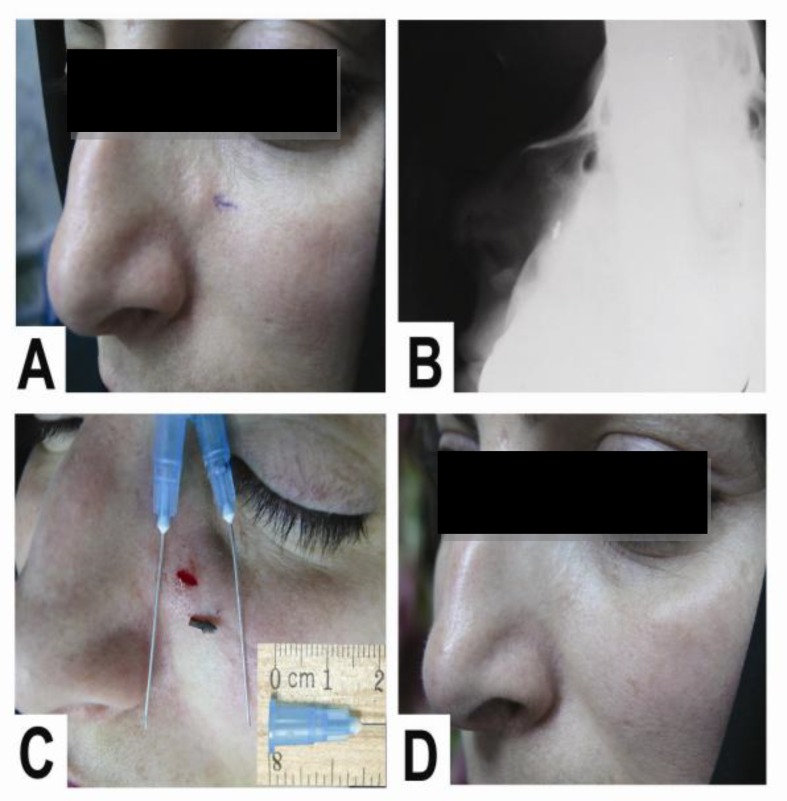
A, 32-year old woman injured 20 years earlier; B, The neglected pen tip as observed radiographically; C, Localization and removal of the pen tip via a small incision; D, Six months after surgery. Note the mild scar formation

We discontinued further blind wound exploration in these three cases. Ecchy- mosis that resolved spontaneously after 5–10 days was observed in three patients after surgery in the periorbital area. Noticeable scars was not observed at all operation sites, 6–12 months after surgery, as shown in follow-up photographs (Fig 4). Pre-and post-operative neurologic and functional examinations were identical. 

## Discussion

There are numerous indications for neglected STFB removal based on possible complications such as persistent pain, neurovascular damage, cosmetic, or psychological issues. However, localization and removal of neglected STFBs is complex due to possible inflammation, indurations, granulated tissues, and fibrotic scars (3). There are several possible approaches to the surgical removal of STFBs based on the nature, size, location, and neurologic and functional complications of the body. 

**Fig 5:**
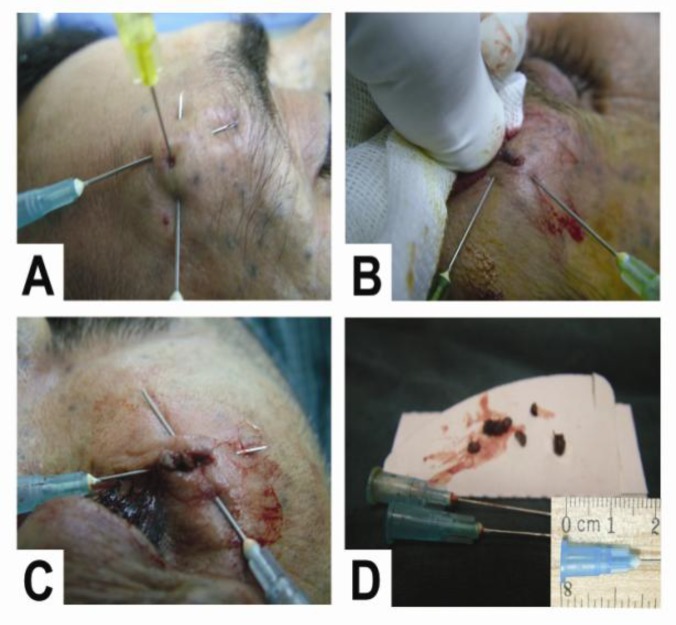
A, 54-year old man with injuries inflicted 24 years earlier. Note the presence of traumatic tattoos due to a mine explosion; B, C and D Note the foreign bodies were surrounded by fibrotic tissues

In this study, the cross-needle-guided STFB surgical technique was successful in 38 of 41 neglected STFBs. This method is a simple office-based technique that allows proper localization, fixation, and propulsion of the foreign body towards the surface of the skin. This surgical approach leads to a reduction in the size of the incision and prevents the foreign body sliding during incision and dissection. Indications for retained foreign-body removal in our patients included persistent pain, local tenderness, inflammation, and cosmetic problems such as laser procedures for scars and traumatic tattoos (Fig 3,5).

All foreign bodies in our patients were identified by palpation or by identifying a point of maximum tenderness, and were located in anatomically safe areas.

Precise identification of STFBs by clinical examination is necessary with this technique, and the most important step in the localization of the small foreign bodies is a meticulous insertion of the first needle. However, the use of local anesthesia before inserting the first 23-gauge needle may lead to tumescence and loss of foreign bodies. Use of topical anesthesia or digital nerve/field blocks is therefore more appropriate before insertion of the first needle. Attempts at surgical removal were unsuccessful in the case of three small foreign bodies with a negative radiograph in the dorsum of the hand and the periorbital area using this technique. We discontinued further blind exploration and dissection in these cases to prevent any unwanted trauma to the adjacent structures. 

It is recommended that localization techniques such as ultrasonography and fluoroscopy are used if the foreign bodies were embedded deeply or not easily identified by clinical examination, or if they are located close to a vital structure or associated with neurologic or functional complications. Ultrasonographic detection and guided removal of SFTBs have a sensitivity of approximately 90%, but results show a discrepancy based on the size and nature of STFBs, experience of the operator, frequency of the transducer, and resolution of the image. False-negative results occur when ultrasonography is used for the detection of small STFBs over echogenic structures(12-15). Although ultrasonography is a reliable and accessible technique for the detection, localization, and guided removal of radiopaque and radiolucent foreign bodies, accurate assessment of STFBs depends on use of a high frequency transducer as well as an expert operator who is familiar with the ultrasonographic appearance of STFBs and false positive sources of STFBs such as calcification, scar tissue, fresh hematomas, or air trapped in the soft tissues (19,20). 

There are a number of potential complications with a surgical approach to STFB removal, such as scar formation following enlargement of the wound or creation of a new incision over the STFB, as well as the potential risk of neurovascular injury due to blunt or sharp dissection (5,6,8,9).

A technique has previously been reported in which two small needles are inserted under fluoroscopic guidance into the skin at perpendicular angles until they are touching the foreign body. An incision is then made between the two needles and the foreign body removal is achieved by blunt dissection (5). Although fluoroscopy is useful in the removal of opaque STFBs and may reduce the exposure time to radiation, the technique requires the use of expensive equipment. Furthermore, the perpendicular needles used cannot fix and propel the STFBs toward the skin surface during surgery. Making an elliptical incision around the entry wound and removing the foreign body with surrounding skin and soft tissues is another office-based procedure (4). Tumescence induced by local anesthesia complicates the blind dissection of an old scar without accurate foreign body localization and fixation. The principle disadvantages of these office-based approaches are the likelihood of failure to remove the foreign body and the potential risk of injury to adjacent structures due to the absence of prior localization and fixation of STFBs and excessive blind dissection (5,8,9-11).

In our study, scar formation was minor and no neurovascular or tendon injury was observed (Fig 4D). In STFB surgery, information regarding the location of the foreign body in the soft tissue is achieved by use of plane anteroposterior and lateral view radiographs using radiopaque indicators (16,18); however, radiolucent and small radiopaque foreign bodies may not be detected through this technique. Furthermore, tumescence induced by local anesthesia and sliding of a non-fixed foreign body during surgery may lead to misdirection of the dissection. In our study, 34 of the foreign bodies removed were glass pieces that were small in size (approximately 2–5 mm). Interestingly, the false-negative rate of radiographs for the detection of glass in soft tissue was reported by Levine and colleagues as 25% (3). In contrast, attempts at surgical removal were successful in nine of 12 small (approximately 2–3mm) glass pieces with a negative radiograph using a needle-guided technique. We believe that larger foreign bodies are more detectable by clinical examination, radiography, or ultrasonography and are removed usually in emergency departments soon after trauma. Smaller STFBs, by contrast, are easily missed by clinical examination or localization techniques and remain undetected. In addition, small wounds affected by foreign bodies are not the primary concern of healthcare professionals in patients with multiple traumas and serious life-threatening conditions. Finally, it is important to remove STFBs with minimal dissection and in an atraumatic manner, especially in the face and exposed areas that are cosmetically important for patients. 

## Conclusion

The surgical removal of neglected STFBs using a cross-needle-guided technique is not only a simple office-based procedure, but may also be considered a cost-effective, time-saving and efficient technique which could be used by dermatologists and otolaryngologists for the proper localization, fixation, and propulsion of STFBs towards the surface of skin. Although this remain to be proven, the authors believe that localization of deeper embedded foreign bodies through the cross-needles technique under ultrasonographic or fluoroscopic guidance may further facilitate surgical removal of deeper STFBs and reduce the exposure time to radiation.
